# Effects of Positive Psychology Interventions on the Well-Being of Young Children: A Systematic Literature Review

**DOI:** 10.3390/ijerph182212065

**Published:** 2021-11-17

**Authors:** Valérie Benoit, Piera Gabola

**Affiliations:** 1 Department of Special Education, University of Teacher Education, 1014 Lausanne, Switzerland; 2 Department of Development from Childhood to Adulthood, University of Teacher Education, 1014 Lausanne, Switzerland; piera.gabola@hepl.ch

**Keywords:** positive psychology interventions, early childhood, well-being, health prevention and promotion, review

## Abstract

Over the last 20 years, the effectiveness of positive psychology interventions for the development of the well-being of children and adolescents and the moderation of high levels of anxiety and depression in this population has been largely demonstrated. Emphasis has been placed on the promotion of well-being and prevention of mental health problems in the school context in order to foster, through positive psychology, the cognitive and socio-emotional development of primary and secondary students, e.g., by strengthening positive relationships, positive emotions, character strengths, optimism, and hope. However, little is known about the impact of these interventions on young children. This systematic review aims at examining the effects of positive psychology interventions on the well-being of early childhood children (<6 years old), both in the preschool education context with educators or teachers and also in the family context with parents. Several electronic databases were searched, and the findings systematically reviewed and reported by the PRISMA guidelines. Very few studies met the inclusion criteria (n = 3), highlighting the need for further research in this area. Indeed, all of the selected studies demonstrated the importance of positive psychology interventions with young children to promote positive aspects of development, such as gratitude, positive emotions, life satisfaction, accomplishment, positive relationship, or self-esteem. Limitations in the field are discussed.

## 1. Introduction

Children’s well-being is often associated with objective aspects such as demographic and socio-economic characteristics [1]. In particular, poverty (lack of basic material resources, limited access to health care, and lack of family time) is known to affect, as are other environmental and social issues, youth’s physical and mental health (see, e.g., [2]). It could also affect happiness, another indicator of well-being, in multiple ways by directly or indirectly involving the child [3] (p. 336). In addition to objective aspects, well-being can also be defined more broadly as a subjective experience, i.e., the perception that individuals have of how well their lives are going (see, for example, [4], p. 295). In the field of positive psychology, Seligman [5] indeed defines well-being as “the positive evaluation that people make of their lives” and it “includes positive emotion, engagement satisfaction, and meaning” [6] (p. 1). Positive psychology considers well-being from two perspectives: first, hedonic or subjective well-being is related to positive affect (e.g., happiness), experiences of negative emotions (e.g., trauma and loss of a person), and life satisfaction [7]. Second, eudemonic or psychological well-being is operationalized with positive relationships, autonomy, and purpose in life [8]. Theses perspectives have implications in terms of measuring, despite the most popular measures of well-being are those that assess overall satisfaction or happiness in life [9]. Although it is rare to find studies on the development of programs that include subjective or eudemonic well-being for children at the school or preschool level, positive psychology emphasizes the importance of creating constructive living environments (e.g., families or institutions) throughout the life cycle. The promotion of healthy environmental systems, particularly healthy school environments, is essential to promoting the well-being and mental health of children and young children [10]. Aspects of social and emotional developmental are no less important for children’s well-being and mental health. Thus, even before entering school, skills such as self-confidence, developing positive relationships, and expressing emotions effectively are essential for the future development of young children [11]. A study including 52 systematic reviews and meta-analyses endorses the importance of promoting well-being and preventing mental health problems in schools through early intervention with young children, especially in areas that develop emotional and social skills [12].

Currently, there are many interventions aimed at developing and adjusting emotional and social skills in school, such as social and emotional learning (SEL) programs (see e.g., [13]) or positive youth development (PYD) interventions (see e.g., [14]). However, there seems to be a need to integrate or add other programs whose primary aim is to improve young children’s mental health by targeting well-being as a motivating factor to feel competent in future learning, reducing stress and increasing life satisfaction. Research in the field of positive psychology, the science of well-being and the study of optimal human functioning [15], aims in particular at evaluating practices that can improve well-being in the human life cycle [16].

The research community in positive psychology focuses specifically on positive emotions and positive character traits to improve mental health and promote well-being. The experience of positive emotions contributes to the development of social, physical, intellectual, and psychological resources [17] in adults and adolescents, and also in children. Indeed, the foundations of emotional development are set during childhood and influence the emergence of social and cognitive processes during this period [18]. Positive character traits such as optimism [19] are a quality in youth and adults, and also a potentially beneficial quality for child development [20]. Teaching optimism to children can prevent anxiety and depression problems [21]. Research has also shown that children with higher levels of hope, another positive trait, are more positive about themselves and less depressed than children with lower levels of hope are [22].

In order to test practices that can improve well-being, researchers in positive psychology use placebo-controlled trials of positive psychology interventions (PPIs). These are programs based on practices consistent with positive psychology theory to promote sustainable well-being [23]. Positive psychology interventions are often based on Seligman’s [24] PERMA model, which refers to five conditions: *positive emotions,* which refer to feelings that motivate human actions, such as happiness, pleasure, and optimism; *engagement*, which refers to participation and concentration (flow state) in learning activities; *relationships*, which refers to the perception of having positive and secure relationships, receiving support and appreciation; perceived *meaning*, which involves being able to use one’s strengths to accomplish a goal that is important to oneself; personal *accomplishment*, which refers to the feeling of having achieved personal goals. This model has been adapted to the educational context with the incorporation of a sixth condition, *health*, generating the PERMA(H) model [25]. *Health* refers to optimal physical, emotional, and psychological health, which are crucial aspects of well-being, especially in creating good habits at an early stage for long-term health benefits [25,26]. According to this model, well-being consists of fulfilling one or more of these dimensions, measured independently of each other, which are essential not only for well-being but also for fostering positive affect, the development of secure relationships, life satisfaction, and reducing the risk of mental health problems, including in young children. While the use of these dimensions is a useful and beneficial way to explore well-being, it is also true that while much has been done to depict the dimensions of well-being, there is little to no consensus in the literature on the definition of well-being [27,28,29,30]. “Well-being lacks definition, both as a concept and in practice. Thus there emerges a range of factors identified as inherent in it or against which it is recognizable and/or measurable” [29] (p. 183). In order to go beyond the dimensions and descriptions of well-being in previous research, Dodge et al. [30] proposed a new definition of well-being, which is intended to be simple, universal, and as close as possible to the need for equilibrium or homeostasis of any individual. Thus, they define “well-being as the balance point between an individual’s resource pool and the challenges faced” (p. 230). Another strength of this definition is its optimism, which links it to positive psychology. In this sense, individuals are perceived as agents of their happiness or well-being, who can act on their resources and challenges to maintain a certain state of balance [5,30]. This self-management can be learned and taught, and this is also what positive psychology intervention programs aim to do when they seek to develop or promote well-being, particularly from elements such as those represented in the PERMA(H) model.

### 1.1. Background on the Effects of PPIs

Several meta-analyses conducted with adult samples [31,32,33,34,35,36], with younger people, mainly from 8 to 18 years old [37,38,39], or with both [40] have reported PPIs’ effect on participants’ via increased levels of well-being and decreased levels in depressive, anxiety and stress symptoms compared to the control group. These meta-analyses were conducted with clinical and nonclinical samples, in Western and non-Western countries. Significant small-to-medium effects of PPIs on increasing strengths and quality of life were reported by Carr et al. [40]. Some of these meta-analyses [31,32,38] also reported that findings remained significant after follow-up (two to twelve months), such as decreased depressive symptoms or increased well-being in adults as well as in young people. In terms of moderator impacts (e.g., age, clinical status, and program duration), Carr et al. [40] found notably that PPIs’ effects were higher on quality of life within younger samples and on well-being within older samples. Likewise, Sin and Lyubomirsky [35] reported that age was one of the factors that influenced the effectiveness of the intervention, along with depression status, self-selection, and format and duration of the interventions. In line with Bolier et al. [31] and Sin and Lyubomirsky [35], Carr et al. [40] also showed greatest advantages when PPIs were provided in a long-term individual or group therapy format in clinical population. On the contrary, in a nonclinical population, in an educational context or in another context and with a group or individual, findings indicated that brief PPIs were more effective [40]. Some of these results need to be considered in light of certain limitations, primarily the bias associated with small sample sizes. Indeed, White et al. [41] reanalyzed two highly cited meta-analyses that examined the effectiveness of PPIs on well-being and depression, namely, Bolier et al. [31] and Sin and Lyubomirsky [35]. By taking into account the small-sample-size bias, the results of White et al. [41] indicated that the effectiveness of PPIs on well-being was smaller than the effects found, in particular, by Bolier et al. [31] and, to a lesser extent, by Sin and Lyubomirsky [35], but still significant. In contrast, their results also indicated that “the effects of PPIs on depression were variable, dependent on outliers, and generally not significant” (p. 1). In line with other researchers arguing for preliminary power analysis to establish an appropriate sample size (e.g., [32,40]), White et al. [41] called for more PPI research with a larger sample size.

While positive psychology emerged in the 2000s, its application in the form of positive education and positive intervention in schools arrived later [42]. PPIs in schools are many and different, but they share a common goal: to improve the developmental trajectory of young people and prevent possible future difficulties by teaching positive behaviors [43]. Benefits of positive psychology interventions in schools have been demonstrated notably through systematic meta-analyses [43]. For instance, Brunwasser et al. [38] indicated significant effects of a resilience intervention (Penn Resiliency Programme, PRP) on young people’s levels of depressive symptoms (but not on depressive diagnoses), i.e., lower levels, especially at the post-test follow-up measurement point. On the contrary, the meta-analysis by Bastounis et al. [37], conducted to evaluate the effectiveness of a resilience intervention (PRP) to prevent depression and anxiety in students aged 8–17 years, showed that there is no evidence that these programs and derivatives reduce depression or anxiety. Likewise, although the meta-analysis by Renshaw and Olinger Steeves [39] provided evidence that gratitude is an indicator of subjective well-being in young people (e.g., gratitude is positively associated with positive affects or negatively associated with depression), it also reported that gratitude-based interventions are poorly effective. Despite these mixed results, positive psychology interventions have, as with prevention programs [14,44], the advantage of focusing on all students, children or young children, and not just on those with problems. McCabe et al. [45], referring to Meyers and Meyers [46], pointed out that “exercises in positive psychology to teach concepts such as happiness, gratitude, and life satisfaction can be implemented in schools as measures of primary prevention to promote individual growth as well as positive interactions among all students, not just those at risk” (p. 180). Similarly, enjoyment of school work seems to play a more important role on motivation than the role of anxiety [47]. The school context is therefore seen as a key criterion for assessing the well-being of children. Children’s quality of life is also an important indicator reported in international OECD surveys, as poor schooling has later consequences for the adult life course [48]. Finally, a positive school climate has an impact on children’s happiness and well-being at school [45].

### 1.2. Study Objective

With the exception of the meta-analysis by Carr et al. [40], where 20% of the selected studies were conducted on samples of children and adolescents (<18 years old), previous meta-analyses were mainly based on the effects of PPIs in adults. Although three meta-analyses have been conducted on the effects of PPIs in young people [37,38,39], most of the samples are from late childhood or adolescence rather than from early childhood. Given the interest of positive psychology in preventing mental health problems by improving the well-being and quality of life of children, adolescents, and adults, research on this topic targeting children in early childhood seems crucial in terms of promoting healthy living environments, positive habits, as well as in an inclusive perspective [49]. Indeed, early childhood is a fundamental period of emotional and cognitive development. It is therefore considered to be a particularly favorable time for fostering future well-being in life [45]. However, little is known about the benefits of PPIs for young children. Thus, the purpose of this systematic literature review is to examine studies that have demonstrated the effectiveness of positive psychology interventions on the well-being of young children (<6 years old), both in the context of preschool education with educators or teachers and in the family context with parents. It is essential that parents, educators, and teachers have knowledge and understanding of such programs (PPIs) and their effectiveness in order to encourage, in a promotional and preventive perspective, the development of well-being in early childhood. Indeed, “increases in well-being are likely to produce increases in learning, the traditional goal of education”, but also increases in life satisfaction and decreases in depression [42] (p. 294).

## 2. Materials and Methods

The search process adhered to the Preferred Reporting Items for Systematic Reviews and Meta-Analyses (PRISMA) [50]. Literature searches of original studies published from 2000 to 2020 (as of 22 October 2020) were conducted in Web of science (Core Collection), OVID, and PubMed electronic databases. Various keywords were used in the search, including terms describing (1) the research area and the application (e.g., positive psychology intervention or positive psychology program), (2) the evaluation (e.g., intervention effect or efficacy), (3) elements associated with the PERMA(H) model and possible outcomes (e.g., well-being, quality of life, mental health, positive emotion, engagement, meaning, relationship, or achievement), and (4) the target population or setting (e.g., young children, toddler, preschool, kindergarten, or parent).

The inclusion criteria allowed for the selection of only (1) peer-reviewed studies (2) published from 2000 to 2020, (3) written in English, and (4) measuring the effects of (5) a positive psychology intervention (PPI) on (6) the well-being of (7) young children (<6 years old). More specifically, participants could be male or female children, with or without disabilities, with or without physical or mental health issues, and from community or clinical samples. Studies with samples of preschool and older children were also retained. In addition, the interventions were to aim to improve children’s well-being through activities related to positive psychology theory (i.e., PPIs). There were no restrictions on the professional experience of those conducting the interventions, the contexts in which the programs were carried out, or the mode of implementation. Only studies reporting data both before and after the intervention (with or without a follow-up measure) using self-report or clinician/researcher-administered psychometric instruments were retained. Studies with no alternative control groups, as well as studies without control groups were taken into account. Well-being was considered in its broad definition and was associated with at least one dimension of the PERMA(H) model [24,25]. No other limitations in terms of outcomes were imposed.

Exclusion criteria were (1) study sample over 6 years old only (e.g., primary school children, adolescents, or adults), (2) absence of PPI, (3) absence of outcomes, outcomes not reported or qualitative results only, (4) no pre-/post-test assessment, (5) no variable linked to PERMA(H) model, (6) literature review or books, and (7) grey literature (e.g., dissertations or conference proceedings). Studies on positive parenting programs (i.e., Triple P) have also been excluded as their efficacy has already been widely demonstrated (e.g., [51,52]). The screening process was conducted in Excel sheets; no automation tools were used. For the entire process, the two authors discussed discrepancies between their respective independent evaluation until they reached consensus.

In line with inclusion criteria, data extraction was carried out using the PICOTS method [53] and performed in a double-blind manner by listing, in an Excel sheet, the following categories: authors, year of publication, country, study objective, population characteristics (age, gender, and developmental characteristics), intervention (type and description), comparator (e.g., control group), method used, assessment instruments, time, setting, and outcomes in terms of effects of positive education intervention programs on children’s well-being. Thus, outcomes were subdivided with the PERMA(H) model dimensions (i.e., positive emotions, engagement, relationships, meaning, accomplishment, and health) [24,25]. The health dimension has been subdivided into five categories: mental health (i.e., behavior problems), emotional well-being, global well-being, self-esteem, and life satisfaction. The discrepancies were discussed and recoded by both authors in order to reach full agreement.

## 3. Results

### 3.1. Identification of Eligible Studies

**Figure 1:**
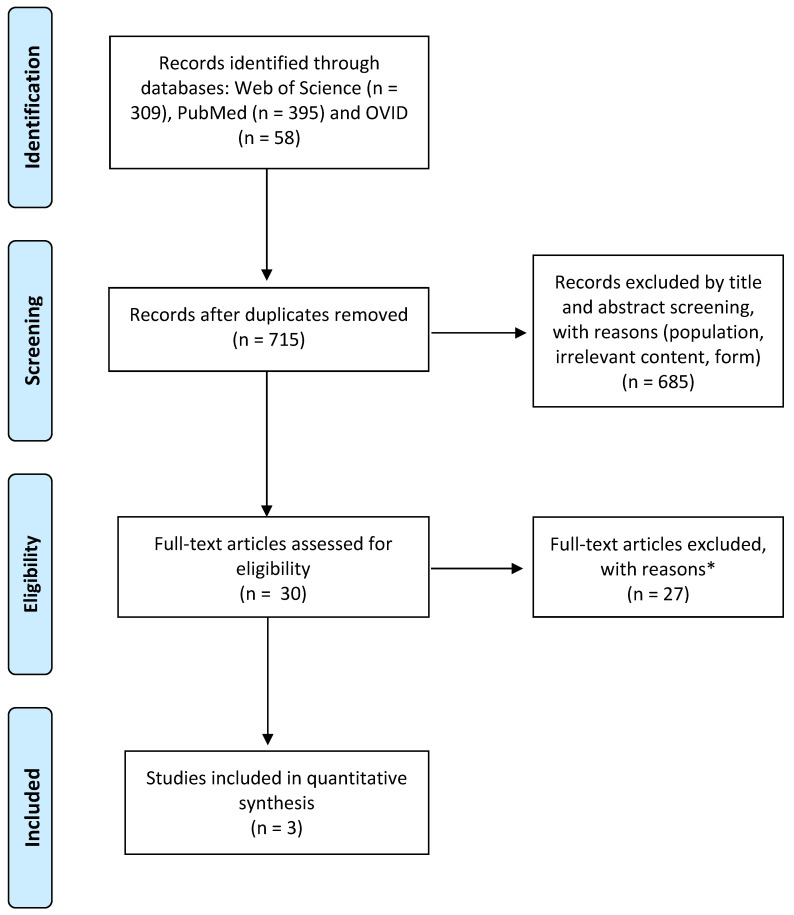
PRISMA flow diagram. * Reasons for exclusion included ineligible population (n = 15), ineligible intervention (n = 9), outcomes not reported (n = 3), other (irrelevant content, n = 1; ineligible language, n = 1; inadequate methodology, n = 1). Adapted from Moher et al. [54].

As presented in the PRISMA flow diagram (Figure 1), the initial literature searches returned 762 papers, and 715 results remained after removing duplicates. Title and summary screening led to the removal of 685 articles not meeting the inclusion criteria, mostly due to population ineligibility (>6 years old), nonrelevant content and form (e.g., conference paper, review, language other than English, or incorrect reference). A total of 30 studies were included by the title and abstract screening for text eligibility. Discrepancies in eligibility were discussed between the authors until a consensus was reached. Finally, 27 publications did not fit the inclusion criteria (e.g., population characteristics, absence of PPI, review or theoretical, or no English language), and only three studies remained for review.

The following sections present the detailed results according to the PICOTS method for intervention studies reviews [53]. The population of the studies is described first, followed by the characteristics of the interventions, including information on comparator, time, and setting. This is followed by details on key methods of the studies (e.g., research design and assessment instrument). Finally, results in terms of the effects of positive psychology interventions on young children’s well-being are reported using the variables related to the PERMA(H) model.

### 3.2. Populations of the Studies Reviewed

Table 1 provides an overview of the selected studies in terms of country, sample, and intervention characteristics. The selected studies were conducted in three different countries (Netherlands, the USA, and Israel) and published in the last decade (2013 to 2020). Sample characteristics indicate that only one study included a specific sample of young children (<6 years; 315 children aged 3–6 years [55]). The other two included a broader age range of 4–12 years [56,57]. Results of Elfrink et al. [56] are discriminated for children from grade 1 (4–5 years) to grade 3 (6–7 years), with n = 32 remaining after parental permission, but not for preschoolers in grades 1 and 2 only. No such distinction is provided in the study by Owens and Patterson [57]. This finding reflects the current paucity of research focusing solely on preschoolers. Only one study included other populations in its sample, namely, parents and school staff [56]. In all selected studies, the ratio of girls to boys is well balanced (see Table 1). Finally, all three studies used a community sample without mentioning or focusing on children with special educational needs.

### 3.3. Interventions, Comparators, Time and Research Settings

The positive psychology interventions of the three selected studies are very different in terms of content, duration, target population, and settings. An overview of the PPI characteristics is described in Table 1. Their commonalities are highlighted further below.

In terms of setting, and as they did not target a unique preschool population, Owens and Patterson [57] recruited participants from suburban schools of middle- to lower-middle-class population. Elfrink et al. [56] selected two schools (rural/urban) in Netherlands in line with previous recommendations of “whole-school approach to positive education for primary schools” (p. 216). Shoshani and Slone [55] selected 12 demographically similar preschool classrooms in a central city in northern Israel (same geographic area). Regarding duration, one study [57] was conducted over a few weeks, comprising four to six intervention sessions. The other two studies [56,57] were conducted over a school year, with a consequently larger number of sessions.

Although only two studies [55,56] explicitly refer to the PERMA(H) Seligman’s [24] model, all three examined how PPIs are applied to the educational context. We can argue that the dimensions studied in each study can fit into one or more areas of the PERMA(H) model. The emotional dimension is taken into account in most studies as an essential aspect of young children’s well-being. As shown in Table 1, the *positive emotion* of empathy is developed in one program [55] and the *positive emotion* of gratitude in two programs [55,57]. *Engagement* was worked on in two of the three programs considered [55,56], *positive relationships* in one program [55] and *accomplishment* in two [55,57]. *Meaning* has not been addressed in these programs.

### 3.4. Key Methods of the Reviewed Studies

With the aim of examining the effects of an intervention, the selected studies all used a longitudinal research design (see Table 2). All included at least two measurement points, pre- and post-intervention, at intervals of a few weeks [57] or several months within the same school year [55,56]. No study included an additional midpoint measurement or a delayed post-test follow-up measurement point. Two of the selected studies opted for a quasi-experimental design. The study by Shoshani and Slone [55] compared 160 children in the intervention condition to 155 children in demographically similar control classes. Owens and Patterson [57] compared three groups: an intervention group (gratitude condition), an alternative intervention group (best possible selves’ condition), and a control group. Elfrink et al. [56] did not include a control group. However, it is the only study to adopt a whole-school approach and to present a mixed-method research design by adding qualitative interviews with parents and school staff to quantitative measures.

To measure outcomes associated with children’s well-being through variables related to the PERMA(H) model, a number of reliable assessment instruments were used (Table 2), either self-reports in individual interviews with the researchers or parent report: The Positive and Negative Affect Scale for Children (PANAS-C [58,59]) [55,57], a modified version of the Brief Multidimensional Students’ Life Satisfaction Scale (BMSLSS [60]), the Affective Situations Test for Empathy (FASTE [61]), the Perceived Competence Scale for Children (PCS-C [62]) to measure self-esteem, and the Kiddy Health-Related Quality of Life for children questionnaire (KINDL-R [63]) to measure children’s well-being. Shoshani and Slone [55] also assessed children behavioral regulation with the Head-to-Toes task (HTKS [64]) as well as children’s learning behaviors with the Approaches to Learning Scale (ALS [65]). Some instruments were modified in several ways to be completed by children, particularly preschoolers (e.g., items were read aloud; wording was simplified; the response scale was reduced; the Likert scale was pictured; see, e.g., [55]). The student–teacher relationship was completed by teachers in Elfrink et al.’s [56] study with the Leerkracht Leerling Relatie Vragenlijst (LLRV [66]). Finally, two of the selected studies [55,56] also assessed outcome variables related to the health dimension, namely, children’s emotional and behavioral functioning (or mental health), measured with the Strength and Difficulties Questionnaire (SDQ [67]) parent report form.

### 3.5. PPIs’ Effects on Preschool Children’s Well-Being

The results of the selected studies are summarized in Table 2. They are presented according to the PERMA(H) model, with the exception of the dimension meaning, as none of the selected studies reported outcomes on it.

#### 3.5.1. Positive Emotions

Two studies examined the impact of PPIs on positive and negative affect [55,57]. Their results differ slightly. Owens and Patterson [57] found no effect on positive affect in any condition (i.e., gratitude, best possible selves condition, or control condition). However, they showed that children as young as 5 or 6 years old were able to experience and to express gratitude for a variety of people or events (i.e., through drawing and explaining what they were grateful for).

Although neither Owens and Patterson [57] nor Shoshani and Slone [55] found any effects of the PPIs on negative emotions, Shoshani and Slone [55] found a significant increase in positive emotions after the intervention in the experimental group, especially empathy, as reported by the children themselves and their parents.

#### 3.5.2. Engagement

The positive impact of the PPIs on children’s engagement in classroom activities is supported by qualitative evidence reported by teachers in the study by Elfrink et al. [56]. Teachers also reported how the program led them to improve their ability to observe and support students’ engagement. Similarly, teachers in the study by Shoshani and Slone [55] reported a significant increase in children’s engagement in the intervention group (medium effect size) but not in the control group. However, they did not find a significant impact of the PPI on children’s self-regulation.

#### 3.5.3. Relationships

Shoshani and Slone [55] reported a significant increase of pro-social behaviors in the intervention group, but not in the control group. Although pro-social behaviors can lead to more positive relationships, no significant change in the overall teachers’ perception of his or her relationship with the students was found between pre- and post-intervention in Elfrink et al.’s [56] study. However, subscale scores revealed that such PPIs lead to significant improvements in teachers’ closeness with students, including experiences of enhanced affection, warmth, and communication. In addition, in this study, parents reported a positive impact of the intervention on the overall school climate (large effect size), with a significant improvement in subscale scores such as “supportive cooperation and active learning”, “forbidding physical punishment and violence”, “not tolerating bullying, harassment and discrimination” and “promoting equal opportunities and participation in decision-making”.

#### 3.5.4. Accomplishment

This dimension is examined very differently in two of the selected studies. Elfrink et al. [56] reported that the PPIs improved teachers’ awareness of students’ skills and talents, which are known to be critical for achievement. In Shoshani and Slone’s [55] study, teachers reported improved preschool functioning in the intervention group (i.e., positive learning behaviors: enthusiasm for learning, attention, autonomy, and persistence).

#### 3.5.5. Health—Well-Being

Several studies measured the PPIs’ effect on children’s overall well-being. Elfrink et al. [56] reported a positive impact on children’s self-report of well-being, with a larger effect for younger children on health-related quality of life (p. 224). Owens and Patterson [57] as well as Shoshani and Slone [55] measured children’s well-being through life satisfaction. Unlike Owens and Patterson [57], who found no significant effect of the intervention on life satisfaction (regardless of condition), Shoshani and Slone [55] found a significant increase in children’s life satisfaction in the intervention group but not in the control group. Regarding self-esteem, which is often acknowledged as a predictor of later health condition, Owens and Patterson [57] found a significant increase over time in the best possible selves condition, but not in the gratitude or control condition.

Two studies also examined the effect of PPIs on well-being in terms of mental health (i.e., behavior problems) measured with the SDQ. In this case, results also differ. Elfrink et al. [56] noted a positive impact of the intervention on behavior problems, including hyperactivity, emotional problems, and relationship problems. However, although the total difficulty score showed a significant decrease between pre- and post-intervention (medium to large effect size), no significant changes were found in the subscales. These quantitative results were confirmed by qualitative evidence from teachers about the positive impact of PPI on children’s positive behavior. Parents also reported a decrease in behavioral difficulties. On the contrary, Shoshani and Slone [55] found no change in mental health difficulties after the intervention. They found a significant effect of time in both the experimental and control groups i.e., a decrease in mental health difficulties.

#### 3.5.6. Moderator Effects

All selected studies controlled for moderator effects. Among different variables (e.g., age, educational level, gender, family, and socio-economic status), only age and gender had significant effects. Shoshani and Slone [55] reported that age was the only variable found to be significantly correlated with one outcome variable (conduct problems). They also found that children’s age was positively related to higher subjective well-being and lower mental health problems at the beginning of their study. As for Owens and Patterson [57], their results indicated age effects on the frequency of realistic possible selves responding (i.e., declining with age). Finally, Elfrink et al. [56] reported larger effects of the PPI on quality of life (health-related variable) in younger children than in older ones.

In Owens and Patterson’s [57] study, gender was found to have significant effects in that girls reported realistic best possible selves more frequently than boys did. Without having a significant effect on the outcome variables, Shoshani and Slone [55] also found a gender difference, with boys having more behavior problems and fewer positive and negative emotions than girls.

## 4. Discussion

The aim of this review was to examine the effects of positive psychology interventions on the well-being of young children (<6 years), both in the context of preschool education with educators or teachers and in the family context with parents. Our review produced only three articles that met our criteria. However, the results of the selected studies provide preliminary support for the usefulness of PPIs in improving the well-being of young children. Before discussing these main findings in relation to the PERMA(H) model, we first present a general discussion based on the descriptive characteristics of the selected studies. The main limitations of our study will then be discussed, and implications for practice and future research will be highlighted.

### 4.1. Overall Discussion

Descriptive characteristics from the three selected studies indicate firstly that empirical interest in the effects of PPIs on young children is relatively recent, with the first published study dating from 2013. More importantly, these studies are extremely rare and have been conducted exclusively in school or after-school settings, but not in family settings. Moreover, the selected studies only included typically developing young children aged 3–6 years, leaving out children aged 0–2 years and children with special educational needs. This is consistent with previous meta-analyses of the effects of PPIs, indicating that positive psychology interventions appear to be most applicable with adults and youth in late childhood and adolescence, as well as in the educational context, and less so in the family context. In a related area, Renshaw and Olinger Steeves [39] also reported a very small number of studies examining the effects of gratitude-based interventions among young people (i.e., n = 5). However, several explanatory hypotheses deserve to be addressed. 

First of all, one of the necessary conditions for implementing PPIs in schools and other educational settings (e.g., daycare) is to have trained educators, notably because they can be the connection between school and families [68]. For parents, being trained allows them to contribute and reinforce at home the message that children learn at school [12]. However, positive psychology is not yet well developed in the initial training of teachers or educators, implying that researchers in this field have to train participants, which takes time and resources. The intervention design of two of the selected studies [55,56] in our review included teacher and/or parent training in positive psychology or related area. Training in positive psychology prior to or concurrent with the intervention led to several advantages. Elfrink et al.’s [56] qualitative results indeed indicated that both teachers and parents positively assessed the professional workshops they attended. In particular, teachers reported the importance of training focusing on practical strategies, guidelines, and activity-based resources to support the roll out (and the perpetuation) of positive psychology interventions (p. 225). The results of this pilot study also showed that over the course of the school year, the positive education program was gradually integrated into daily school activities and teachers were better able to understand their role in the program and to continually provide children with positive psychology-based activities. This highlights the importance of ongoing training for adults in such programs as well as the continued integration of positive psychology practices into daily school activities and routine educational practices, as has already been demonstrated for SEL programs [69,70]. However, more than specific knowledge and teaching strategies acquisition, there is a strong need for changes in educators’ attitudes, beliefs, and values [12]. 

Secondly, the lack of research on the effect of PPIs on the well-being of young children may be because preschools and kindergartens place a disproportionate emphasis on the cognitive aspects of being ready for school, while it would also be important to focus on well-being aspects (e.g., social and emotional aspects) from an early age [71].

Thirdly, this may also be due to the fact that measuring the effects of PPIs involves the administration of self-report questionnaires before and after the intervention. Yet, as reported by Park and Peterson [3], self-report questionnaires are a limitation in research with very young children due to their level of language development and cognitive maturation. Similarly, the aspects of “meaning” are difficult to include in interventions with young children [72] if researchers are to apply all of the five conditions of the PERMA model to assess well-being.

Gratitude interventions are also questionable in young children, despite Owens and Patterson [57] finding that children under 7 years of age are cognitively mature enough to experience and express it. Indeed, gratitude appears to be a process that is developing over several years and consolidating by mid-childhood [73], making the effectiveness of gratitude interventions unclear in the scientific literature, particularly for younger children [39].

Finally, another possible explanation for this lack of studies on the effects of positive psychology interventions on young children’s well-being is that positive psychology is a recent scientific discipline (emerging in the early 2000s). Chodkiewicz and Boyle [43] also point out that “it will not be until the discipline has matured and researchers are able to carry out more comprehensive and longitudinal research studies, along with extensive meta-analyses, that the research field will begin to see the full potential of school-based positive psychology programmes” (p. 72). In line with this hypothesis, the paucity of PPIs for young children can be due to the already widespread use of SEL programs [70], which also provide systematic training in preschool settings on how to support children’s social-emotional development and improve their self-regulation skills.

After these initial considerations, the following discussion is intended as a reasoned interpretation of the results obtained due to the main limitations of the study outlined hereafter.

### 4.2. PERMA(H) Outcomes

As mentioned in the introduction, the difficulty in finding a consensus definition of well-being can present significant barriers to the implementation and evaluation of programs aimed at improving well-being in schools (see, for example, [74]). Although the dimensions of the PERMA(H) model do not define well-being per se, they nevertheless represent constituent and measurable elements of well-being [30,75]. Indeed, these dimensions allowed us to not only select articles related to both positive psychology and well-being, but also to organize and aggregate research findings in this area. 

Our findings on the PERMA(H) model outcomes point out that all different kinds of programs lead to positive effects on children’s overall well-being. Specifically, with regard to *positive emotions,* two studies [55,57] examined the impact of PPIs on positive and negative emotions. Yet, an increase in positive emotions in the experimental group was found in only one study [55], highlighting the potential of these programs to promote positive emotions in young children, but also its inconsistency. A possible explanation for this increase in positive emotions in the study by Shoshani and Slone [55] but not in Owens and Patterson’s [57] study relates to the different duration of the intervention, with the former lasting much longer (months) than the latter (weeks). While this is not consistent with the findings of Carr et al. [40] that, in general, short PPIs are more effective particularly in educational settings, it is consistent with the findings and recommendations of Weare and Nind [12] regarding school-based interventions of all kinds that promote child well-being. Another explanation for this discrepancy may lie in the nature or the format of the intervention. In the study by Shoshani and Slone [55], the intervention was run by preschool teachers, equipped with a manual containing practical and theoretical material on four modules addressing different themes, including a specific one on positive emotions. This is interesting considering that, as Villarreal et al. [76] reported, knowledge of the theory and usefulness behind a program positively influences teachers’ engagement in its implementation. In contrast, in Owens and Patterson’s [57] study, the intervention was managed by a research assistant. It could therefore be that teachers who are in constant contact with the pupils and have a strong relationship with them can influence students’ positive emotions more than a research assistant who barely knows the children can. Furthermore, teachers have the opportunity to be in contact with students even on school days when the intervention is not taking place, thus promoting the skills acquired during the intervention on an ongoing basis [77]. Finally, no significant effect on negative emotions was observed in the study by Shoshani and Slone [55]. According to the authors, this result can be explained by the duration of the program, which does not seem to be sufficient to learn how to manage negative emotions for such young children. Indeed, at this stage of life, the regulatory system is not yet developed. These are probably the same reasons why, in Owens and Patterson’s [57] study, gratitude interventions did not influence children’s negative affect.

Regarding the dimension of *engagement,* results from two studies showed that children’s engagement in school was reported by teachers to be improved by the intervention [55,56]. Although Elfrink et al. [56] did not find results using a control group, their findings are consistent with those of other studies that have also shown how implementing a school-wide positive education approach has positive effects on improving school engagement, as well as achievement and health [2,78]. This effect of PPIs is interesting considering (1) that preschoolers’ positive engagement promotes better attention and impulse control [79,80] and (2) that pupils’ cognitive and behavioral skills at the time of school entry can predict school engagement some years later [81,82]. Moreover, a continued focus on engagement allows children’s resources to be valued over their limitations, thereby improving their academic performance [83].

In terms of (positive) *relationships*, Elfrink et al. [56] found no significant improvement in the overall teacher–student relationship, but they did report a positive impact of the program on one subscale, namely, teachers’ closeness to children. Children’s behavior is notably predicted longitudinally by the quality of the teacher–child relationship [84]. Specifically, teachers who establish positive emotional connections with children create an environment that is conducive to children’s ability to self-regulate their behavior. The demands and supports available (e.g., provided by the teacher) within a preschool classroom also influence children’s ability to regulate their behavior, emotions, and thoughts [84]. Furthermore, Elfrink et al. [56] found a positive impact of the intervention on improving supportive cooperation, promoting equal opportunities, and participation in decision-making, elements that promote positive relationships. Their intervention also led to improvements in behaviors such as the prohibition of physical punishment and violence and the nontolerance of bullying, harassment, and discrimination. 

Findings on *accomplishment* were found in two studies [55,56]. In the school context, this dimension includes achievement in different school areas [25]. Elfrink et al. [56] reported that teachers were more aware of students’ talents. Teachers in Shoshani and Slone’s [55] study reported greater positive approaches to learning (e.g., enthusiasm for learning, attention, persistence, and autonomy) among children in the experimental group than in the control group. Yet, attention and perseverance would have a mediating effect on the relationship between cognitive flexibility and school readiness [85]. According to Shoshani and Slone [55], a better preschool functioning “forms the base for a sense of achievement and acquisition of personal goals” and “lays the foundation for learning skills and engagement with learning, which are important qualities that will influence subsequent academic success” (p. 8). Similarly, these strengths are expected to enhance students’ flourishing in school, thereby increasing their satisfaction with school [86]. Achievement through the implementation of these positive psychology programs from an early age can be developed by working and reinforcing children’s talents (e.g., in music, drawing, or sport), thus paying more attention to the potential than to limitations of the pupils.

With regard to the *health* dimension, PPIs target health promotion by addressing quality of life and life satisfaction already in young children. In this respect, one of the selected studies showed significant increases in children’s self-report of life satisfaction [55] and another found a positive impact on younger children’s self-report of health-related quality of life [56]. In our view, these results enhance the likelihood that children who have participated in a positive psychology intervention will have positive emotional and social development as adults. This is also in line with the findings of Weare and Nind [12], who recommend that school-based interventions that promote mental health and prevent problems in schools start early with the youngest children, notably in order to develop social and emotional skills.

### 4.3. Key Limitations

Despite the large number of studies screened, the main limitation is the small number of studies that were ultimately selected (n = 3). This obstacle could have been overcome by conducting the literature search with other, less usual electronic databases, by including grey literature, or by using more creative search terms [87] (p. 239). However, as pointed out for meta-analyses [87], we argue that a very small number of studies selected for a systematic review can transparently indicate the empirical status of a research area. Indeed, it definitely highlights the need for further research on this topic, with young children (<6 years old) and especially in family and day-care settings. Moreover, when very few studies are selected and these have very different characteristics, the synthesis of results may be undefendable [87] (p. 241). Although this may be the case for our review, the numerous and precise inclusion and exclusion criteria and the use of a theoretical model (PERMA(H)) in the conduct of the literature review and in the extraction of data helped to present interesting results in a most transparent manner.

Another limitation is due to methodological concerns in the selected studies. For instance, one study did not use a control group, some have small sample size, and none of them used a delayed post-test follow-up measurement point. We can also point out the lack of assessment tools validated within children samples. In addition, studies including preschool and older children did not clearly identify preschool subsamples (<6 years old), which would allow the presentation of results differentiated by children’s age. Indeed, the effects of PPIs on young children may differ from those of older children, depending on the former’s ability to understand certain concepts (e.g., gratitude; [57]) or their ability to see things in a systematically positive way [56,63].

### 4.4. Practical Implications

The diversity of PPIs implemented in the three selected studies allows us to present some useful practical recommendations. Firstly, according to Elfrink et al. [56] and as discussed above, the implementation of PPI in educational settings needs to be combined with training for educators in order to have a synergistic effect on children. This training should address a number of issues, such as the explicit link between theory and practice or the possibility for school staff to “live” the tenets of positive education, i.e., the skills taught within a PPI [25] (p. 151). Thus, the training should provide educators with activity-based strategies and resources to implement theoretical concepts based on positive psychology in daily practice and to integrate them in the curriculum [25]. Training should also be ongoing in schools, especially in response to the specific needs of educators.

A whole-school framework, as in the study by Elfrink et al. [56], is also recommended to improve student well-being [12,31,42,78] and to embed positive education throughout the school system [2,25]. Indeed, this approach not only considers the curriculum and classroom setting, but also shapes the whole school, including its organization, relationships, physical environment, curriculum, and teaching practices [88].

Another practical implication concerns the duration of the intervention. Despite the paucity of selected studies and contrary to the findings of Carr et al. [40], longer interventions appear to be more effective, as has been shown for adult samples [31,35], for other school-based mental health interventions [12] or for intervention programs to improve well-being in the family context [89].

Finally, creative methods like drawing, role-playing, or child-to-adult dictation should be explored as means to implement PPIs [57]. For young children in particular, creative ways to develop character strengths can pave the way for promoting well-being [25].

### 4.5. Future Research

Taken together, our findings suggest that this topic is very poorly studied within young children. Further studies using robust research methods to explore the effectiveness of PPIs with large samples are therefore particularly needed [41,55,56], especially given the importance of schools benefiting from such programs to improve the emotional and social well-being of young children (see also [12]). Moreover, future research could benefit from building on the dimensions of the PERMA(H) model, as they provide a useful and valid conceptual framework not only for implementing practices (PPIs) that promote children’s (and more broadly teachers’ and school community’s) well-being, but also for evaluating their effectiveness, as demonstrated, for example, in Shoshani and Slone’s [55] study.

Authors also called for immediate impacts of PPIs as well as long-term effects on children, as well as on teachers and the school as a whole [25,56]. Monitoring the long-term effects of the seeds of positive education sown in early childhood with longitudinal studies is therefore a necessity [55] (p. 9). However, demonstrating the long-term benefits of PPIs cannot take place without greater monitoring of children’s well-being and increased research support for planned PPIs in educational settings, notably preschool. Thus, research in this area could also follow the example of research on the effects of SEL programs not only on general well-being but also on academic performance to help convince school policymakers and stakeholders to implement PPIs. Indeed, they should be able to see how well-being can benefit the development of academic skills [90] and see it as a complementary rather than a competitive goal [25].

In addition, all three selected studies used a community sample without focusing on children with special educational needs, despite the current international trend towards inclusive education. Although the PPI in Shoshani and Slone’s [55] study is integrative in nature and therefore relevant to children with diverse needs and backgrounds, future research could explore the impact of PPI also on young SEN children explicitly, including those with behavioral difficulties, using community and clinical samples. In comparison, the effects of social and emotional learning (SEL) programs in school, and notably among high-risk children, are already well demonstrated in a variety of areas such as achievement, misbehavior, and mental health (see e.g., [12,69]).

Moreover, it seems essential to construct and validate appropriate measurement tools for young children. In this regard, the adaptations made by Shoshani and Slone [55] are typical examples of avenues to be explored and validated. Finally, given that our results are only from three countries, it would be relevant to explore the effectiveness of these programs on young children’s well-being in other educational settings.

## 5. Conclusions

This article summarized 20 years of research on the impact of positive psychology interventions on the well-being of young children (<6 years). To our knowledge, this is the first systematic review of the literature to examine the effects of these programs on the well-being of such a young population. Despite encouraging results in various areas (e.g., positive emotions, social competency, and positive relationships), our review demonstrated the paucity of research examining positive psychology interventions for preschool children. As mentioned earlier, we believe that it is essential to integrate these interventions into existing programs (e.g., SEL) at an early stage so that they can play a positive role in children’s developmental trajectories. Indeed, early childhood is a time of major acquisitions in many areas, including social-emotional skills [91], which are known to support school readiness, help prevent later mental health issues [92,93,94], and to promote well-being, which in turn should be an indicator of school success [95]. Certainly, talking about well-being in terms of positive psychology (i.e., focusing on children’s resources rather than their limitations, [56]) is a shift in perspective. We are aware that there is much to be done, such as replicating and confirming the few existing studies, before clarifying the findings in the field of positive early childhood psychology [43] (p. 78).

## Figures and Tables

**Table 1:** Overview of the positive psychology intervention programs.

Study	Sample (N; Age; Sex Ratio)	Program/ Intervention	Description	PP Component (PERMA)
Elfrink et al. (2017) [56] Netherlands	N = 184; 4–12 years; 61% boys	Positief Educatief Programma (Positive Education Program; PEP)	Whole-school approach to positive education for primary schools focusing on improving the well-being of children aged 4 to 12 and creating a positive school climate during a school year. Four core components: values, life rules, well-being, and engagement. Activities and lessons based on previous workshops for teachers on values, rules, well-being, and engagement. Regular process for teachers to intervene and adapt strategies if necessary. Parallel meetings with parents to inform them about PEP and how to support their child’s well-being at home.	Engagement
Owens and Patterson (2013) [57] USA	N = 62; 5–11 years (M = 7.35, SD = 1.73); 48.39% boys	Gratitude Sources and Best Possible Selves	Program of 4 to 6 weeks duration (once-weekly sessions) for children from 5 to 11 years old, taking place after school care and summer day camp programs. Small-group intervention sessions (3–10 children per group) where children were asked to draw either what they were grateful for (sources of gratitude—intervention group), a fictional future situation in which they would be at their best (best possible self—alternative intervention group) or something they had done during the day (control group). They were also asked to describe the content of their drawing to a research assistant (verbal descriptions recorded).	Positive emotions (gratitude) Accomplishment
Shoshani and Slone (2017) [55] Israel	N = 315; 3–6.5 years (M = 4.53); 48.5% boys	Maytiv Preschool Program	Activities promoting positive emotions, engagement, positive relationships, and achievement during one school year. Eight modules of basic positive psychology concepts tailored to the developmental characteristics of young children (3 to 6 years old), delivered by trained preschool teachers.	Positive emotions (empathy and gratitude) Engagement Relationships Accomplishment

**Table 2:** Methodological characteristics, measures, and outcome summary of positive psychology interventions on children’s well-being.

Study	PERMA(H) Model Element Measured	Key Methods/Outcome Measures	Outcome Summary (PERMAH Model)
Elfrink et al., (2017) [56] NL	Engagement Relationships Health	Longitudinal (T1/T2; 6 months); no control group Children self-reports of well-being (KINDL-R); teacher report of student–teacher relationship (LLRV); parent report of children’s emotional and behavioral functioning (SDQ) and school climate (PEP-sv).	Positive impact of PEP on children’s well-being and problem behavior; improvement in student–teacher relationship; positive impact on students’ engagement.
Owens and Patterson (2013) [57] USA	Positive emotions Health	Longitudinal (T1/T2, 4 to 6 weeks; once-weekly intervention sessions); quasi-experimental (intervention, alternative, and control group) Individual interviews with children to measure positive and negative affect (PANAS-C), life satisfaction (BMSLSS), and self-esteem (PCS-C).	No effect of the intervention on positive and negative affect and on life satisfaction; outcomes in the gratitude condition do not differ from those in the control condition; participants in the best possible selves’ condition show greater gains in self-esteem than do those in the gratitude or control conditions.
Shoshani and Slone (2017) [55] IL	Positive emotions Engagement Health	Longitudinal (T1/T2; 9 months); quasi-experimental Self-report of well-being (PANAS-C), life satisfaction (BMSLSS), empathy (FASTE), and behavioral self-regulation (HTKS); parent report of children’s well-being (PANAS-C-P), children’s mental health disorder (SDQ); preschool teacher report of children’s learning behaviors (ALS).	Significant increase in the intervention group in children’s positive emotions, empathy, and life satisfaction. No changes in negative emotions or for self-regulation. Increase in pro-social behaviors in the intervention group. No significant changes in total mental health difficulties. Effect of the intervention on children’s approaches to learning with significant increase in positive learning behaviors and engagement in the intervention group. Effect sizes for the magnitude of the significant changes in the intervention group were in the small to large range (0.34–0.81).

## Data Availability

Data are available on request.
